# Region-specific microglial alterations in the acoustic startle circuit in Shank3 mice

**DOI:** 10.1016/j.bbrep.2026.102633

**Published:** 2026-05-18

**Authors:** Xin Ren, Robbert Havekes, Martien J.H. Kas

**Affiliations:** Groningen Institute for Evolutionary Life Sciences (GELIFES), Neurobiology, University of Groningen, Nijenborgh 7, Groningen, 9747 AG, the Netherlands

**Keywords:** Microglia, Autism, Sensory processing, Cortex, Brainstem

## Abstract

Autism spectrum disorder (ASD) encompasses a diverse range of neurodevelopmental conditions characterized by variations in social interactions, stereotyped behaviors, and sensory processing differences. Altered tactile and auditory sensory processing is among the most frequently observed phenotypes in ASD animal models, particularly in Shank3 gene knockout rodents. Previous research has focused extensively on neural activity associated with Shank3 deficiency and sensory dysregulation, but the role of glial cells, especially microglia, has been largely overlooked. Microglia, the central nervous system's primary immune cells, are crucial for regulating neural activity throughout development and adulthood. To address this gap, we used immunofluorescence microscopy to examine microglial morphology, density, and the fluorescence intensity of IBA1 and CD68 in adult Shank3 knockout and wild type mice, focusing on brain regions primarily involved in the acoustic startle circuit, while including the somatosensory cortex as a control region. We examined six brain regions involved in auditory and tactile sensory processing: the somatosensory cortex, central nucleus of the amygdala (CeA), caudal pontine reticular nucleus (PnC), reticulotegmental nucleus (RtTg), inferior colliculus(IC), and cochlear nucleus (CN). Our findings showed a 39% increase in IBA1 expression in the CeA (p = 0.01) and a 13% increase in microglial density in the PnC (p = 0.02). However, we found no evidence of robust microglial activation, as indicated by the absence of morphological changes or alterations in CD68 expression across the examined regions. These results indicate that moderate microglial alterations in the Shank3 mouse model may be circuit-dependent rather than a global phenomenon across all sensory modalities, warranting further investigation into the interplay between glial cells and sensory circuit dysfunction.

## Highlights

• Region-specific microglial alterations were identified in Shank3 knockout mice.
• Increased IBA1 expression in the central amygdala and elevated microglial density in the caudal pontine reticular nucleus were detected.
• Altered microglial profiles were detected in brain regions involved in the acoustic startle circuit.

## 1 Introduction

Autism spectrum disorders (ASD) comprise a group of complex neurodevelopmental conditions characterized by differences in social communication, repetitive behaviors, and in sensory information processing [1]. The etiology of ASD is believed to involve a combination of genetic and environmental factors, which together contribute to the pathogenesis and resultant altered behaviors [2]. Many environmental factors influence gene expression, further complicating the understanding of ASD [3,4].

Genetic sequencing studies have been pivotal in identifying genes associated with ASD. For instance, one study involving 5100 autistic individuals and 6212 non-autistic individuals identified 134 genes significantly related to ASD [5]. Another comprehensive study with a sample size of 35,584, including 11,986 autistic individuals, revealed 102 risk genes associated with the condition [6]. Following these discoveries, various research initiatives have focused on modulating the expression of these ASD-related genes in animal models to investigate underlying mechanisms. Among these genes, SH3 and multiple ankyrin repeat domain 3 (Shank3) has been extensively studied. Shank3, a monogenic risk factor for ASD, is a scaffold protein predominantly localized at the postsynaptic density of neurons and functions as an actin regulator, modulating plasma membrane spreading and cell migration [[7], [8], [9]]. Mutant mice lacking Shank3 exhibit reduced social behavior, increased grooming, and dysregulated sensory profiles, mirroring symptoms observed in humans with ASD [10,11].

Sensory deficits are a core symptom of ASD and are closely linked to other ASD symptoms, highlighting the fundamental role of sensory processing in the disorder [12]. Among individuals with ASD, aversive sensory responses are frequently observed, suggesting impaired sensory gating [13]. Increasing evidence indicates dysregulated sensory processing in animals with mutations in ASD-related genes. For example, Shank3 mutant mice display a strong preference for silence and atypical pitch discrimination compared to wild-type mice [14,15]. Besides these acoustic abnormalities, Shank3 mutant mice also showed dysregulated tactile responses to air puff stimulation [11,16].These characteristics make Shank3-deficient mice a valuable model for investigating the mechanisms underlying sensory processing deficits in ASD.

Multiple brain regions are involved in processing sensory information. Sensory abnormalities in ASD include alterations in both auditory and tactile processing. The somatosensory cortex plays a central role in tactile perception and has been reported to exhibit functional alterations in individuals with ASD [17,18]. In the present study, the somatosensory cortex was included as a comparison region to determine whether microglial alterations observed in auditory-related nuclei represent region-specific changes or reflect more widespread microglial activation. The mammalian auditory pathway consists of several sequential processing stations, including the cochlear nucleus, superior olivary complex, nucleus of the lateral lemniscus, inferior colliculus, medial geniculate body of the thalamus, and the primary auditory cortex. These nuclei form the canonical ascending auditory pathway responsible for transmitting and processing acoustic information from the cochlea to the cortex. Profound defects in acoustic startle response have been observed in Shank3 and other ASD mouse models [10,19]. The mechanisms underlying these defects involve several brain regions and neural circuits. The acoustic startle response is commonly used to investigate auditory processing and sensorimotor integration in rodents. The neural circuitry underlying the startle reflex involves a brainstem pathway in which auditory signals are transmitted from the cochlear nucleus to the caudal pontine reticular nucleus (PnC), which serves as the primary sensorimotor interface generating the startle response. Acoustic information is initially transformed into neural signals in the cochlear nucleus of the brainstem. The reticulotegmental nucleus (RtTg) has been reported to project to the cochlear nucleus and may contribute to the modulation of acoustic startle responses. In addition, the central nucleus of the amygdala (CeA) sends projections to the PnC and is known to regulate the magnitude of startle responses as well as prepulse inhibition(PPI) [20].

While significant work has elucidated the brain regions and circuits involved in sensory processing, the mechanisms of dysregulated auditory processing in ASD remain incompletely understood. Although brain morphological changes and sensory abnormalities in ASD have been reported, there is a lack of evidence demonstrating neurological changes in brain regions specifically related to sensory processing, particularly tactile and auditory stimulation processing.

Microglia, the resident immune cells of the CNS, are involved in neurological repair processes and neuronal network regulation through cytokine release and phagocytosis of misfolded proteins, cell debris, and surplus synapses. Excessive activation of microglia has been implicated in neurological diseases such as Alzheimer's disease, multiple sclerosis, and ischemic brain injury. Numerous molecular markers have been identified to reflect microglial activation, among which IBA1 and CD68 are the most widely used [21]. However, there is limited evidence of microglial activation in ASD brains, especially in regions associated with auditory processing. This study hypothesizes that microglial activation in brain regions related to auditory processing may contribute to the pathogenesis of ASD. We used immunofluorescence and microscopy to measure microglial activation in these brain regions in Shank3 ASD mice. Our findings are consistent with a possible contribution of microglial activation to the dysregulated sensory processing seen in ASD. Based on the known neural circuitry underlying the acoustic startle response, the present study investigated microglial activation in several brain regions involved in this pathway, including the cochlear nucleus (CN), inferior colliculus(IC), reticulotegmental nucleus (RtTg), caudal pontine reticular nucleus (PnC), and central nucleus of the amygdala (CeA). To determine whether microglial alterations are specific to auditory-related circuits, the somatosensory cortex was also examined as a comparison region.

## 2 Methods and materials

### 2.1 Animal

Twelve week old Shank3 KO male mice (B6.Cg-Shank3tm2.1Bux/J, JAX stock #032169) were imported from Jackson Laboratories (Bar Harbor, Maine, USA) to set up heterozygous male by heterozygous female breeding pairs. RT–PCR was performed using Shank3 primers consisting of a common forward primer (5′-AGA TGG CTC AGC CAG GTA AG-3′), a wild-type reverse primer (5′-CAT GTC TCA GTT TGT GCT TGC-3′), and a mutant reverse primer (5′-TGA GAC CAG AGT TGT TAG GAT TTG-3′). Mice were group-housed in standard cages, with 2–3 animals per cage. All mice were bred in-house with a 12-h light/12-h dark cycle under the control of humidity and temperature with free access to food and water. All experiments received approval from the Animal Experiment Ethics Committee at the University of Groningen and were conducted in full compliance with institutional guidelines and EU Directive 2010/63/EU.

### 2.2 Perfusion

Mice at 5 months of age (weighing 25–35 g) were anesthetized with pentobarbital (50 mg/kg) and perfused with 20 mL of 0.9% saline followed by 100 mL of 4% paraformaldehyde (PFA). Brains were extracted and post-fixed in PFA overnight, and then dehydrated by 30% sucrose for 24h.

### 2.3 Immunofluorescence

Brains were embedded in optimal cutting temperature compound (OCT) and sectioned at 20 μm thickness with a cryotome (MNT high-end cryostat, SLEE medical GmbH, Germany). Sections of paired WT and KO mice were mounted directly onto glass slides during sectioning and then air-dried at room temperature for 2 h. The sections were blocked by 5% BSA containing 0.25% Triton-X 100 for 30min. Sections were then incubated in primary antibodies overnight at 4 °C. Then the sections were rinsed by PBS for 5min. After rinsing, the sections were incubated in second antibodies for 1h. Finally, the sections were rinsed 3 times in PBS for 10min per rinse and stained with DAPI. The sections were then covered with the mounting medium (Invitrogen ProLong Glass Antifade Mountant, Catalog number P36984) and cover-slipped. The following antibodies were used: rabbit anti-Iba1(1:200, 019-19741, FUJIFILM Wako Pure Chemical Corporation, rat anti-CD68(1:200, ab53444, Abcam), donkey anti-rabbit Alexa 488(1:200, XJ357262, Invitrogen), goat anti-rat Alexa 555(1:200, 2089884, Invitrogen). For accurate localization of the brain regions, the following stereotaxic coordinates were used based on the *Paxinos and Franklin's Mouse Brain Atlas*: somatosensory cortex (S1) at *AP -1.*5mm, *ML 2.*0mm, *DV -0.*7mm; central nucleus of the amygdala (CeA) at *AP -1.*8mm, *ML 2.*5mm, *DV -4.*3mm; caudal pontine reticular nucleus; inferior colliculus(IC) at *AP -5.*0mm, *ML ±1.*0mm, *DV -0.*7mm; (PnC) at *AP -5.*4mm, *ML* 0mm, *DV -4.*0mm; cochlear nucleus (CN) at *AP -5.*5mm, *ML 2.*5mm, *DV -3.*5mm; and reticulotegmental nucleus (RtTg) at *AP -4.*8mm, *ML* 0mm, *DV -4.*0mm [22]. For the somatosensory cortex and CeA, analyses were performed using sections from the left hemisphere. For PnC, RtTg, IC and CN, tissue from both hemispheres was analyzed.

### 2.4 Imaging

All the images were achieved using a Leica widefield fluorescent microscope. 20X objective lens were used to take the images. Before analyzing, the image files were renamed by several observers in a blind manner. For each brain region, images of 3-4 sections were captured, and all the number of microglia were counted by ImageJ. For the morphological analysis, the branches number, the total and average length of microglia processes by ImageJ. Fluorescence intensity of IBA1 and CD68 was quantified using ImageJ. Images of the corresponding fluorescence channels were converted to 8-bit grayscale. Regions of interest (ROIs) were defined within the same anatomical region in each section. The mean gray value within each ROI was measured to quantify fluorescence intensity. Then the density of CD68 were normalized by the density of IBA1. For brain regions in which both hemispheres were analyzed (PnC, RtTg, IC, and CN), images from the left and right hemispheres were pooled before calculating the animal-level mean. For regions analyzed unilaterally (somatosensory cortex and CeA), only images from the left hemisphere were used. Thus, all statistical comparisons were performed at the level of individual animals.

### 2.5 Statistical analysis

Statistical analysis were performed using GraphPad prism 8.0 (GraphPad Software, Inc., San Diego, CA). Data normality was assessed using the Shapiro–Wilk test. Comparisons between wild-type and Shank3 knockout mice were performed using paired two-tailed Student's t-test. For the threshold of p-value, a number below 0.05 was considered as a significant difference between genotypes. Means ± standard error of mean (SEM) was used to demonstrate the distribution of data.

## 3 Results

**Fig. 1:**
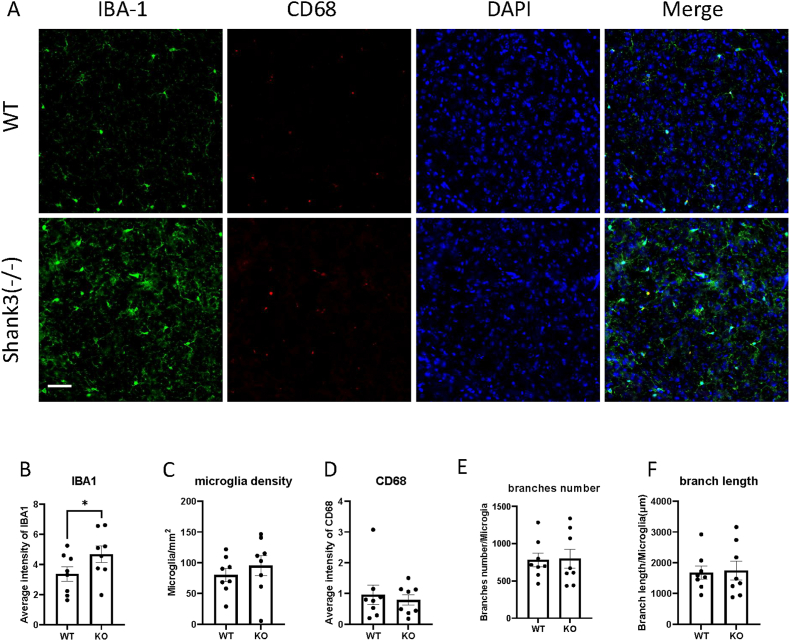
Shank3 knockout mice exhibited increased expression of microglia activation marker IBA1 in CeA. (A) Representative images showing the IBA1 and CD68 expression of CeA in WT and shank3 knockout mice brain sections. (B to F) Quantitative analysis of IBA1 fluorescence intensity(B), microglia density(C), CD68 intensity(D), microglia branch numbers(E) and branch lengths(F) in the CeA of WT and shank3 knockout mice. Scale bars, 50 mm. Data are presented as means ± SEM. *P < 0.05; (WT, n = 8; KO, n = 8).

**Fig. 2:**
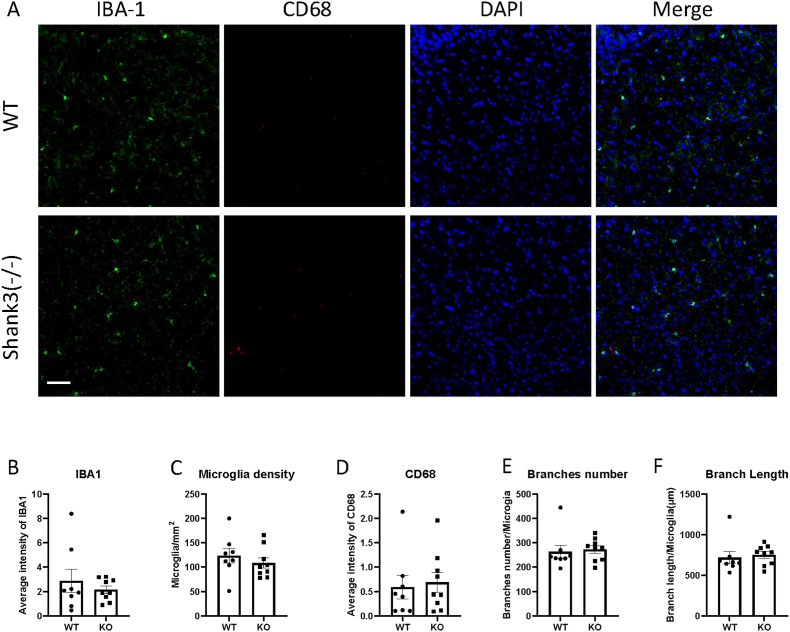
Shank3 knockout mice did not show microglia activation in the somatosensory cortex. (A) Representative images showing the IBA1 and CD68 expression of somatosensory cortex in WT and shank3 knockout mice brain sections. (B to F) Quantitative analysis of IBA1 fluorescence intensity(B), microglia density(C), CD68 intensity(D), microglia branch numbers(E) and branch lengths(F) in the somatosensory cortex of WT and shank3 knockout mice. Scale bars, 50 mm. Data are presented as means ± SEM. *P < 0.05; (WT, n = 8; KO, n = 9).

**Fig. 3:**
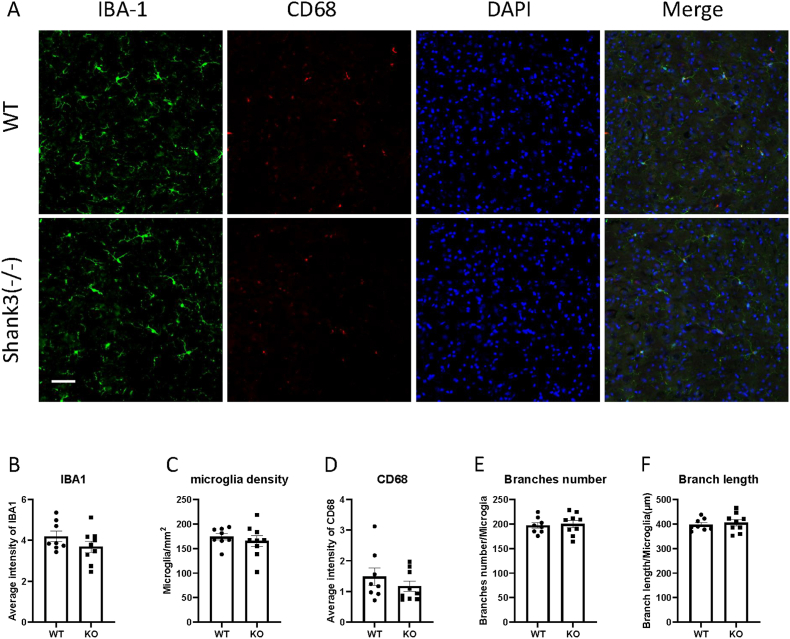
Shank3 knockout mice did not show microglia activation in RtTg. (A) Representative images showing the IBA1 and CD68 expression of RtTg in WT and shank3 knockout mice brain sections. (B to F) Quantitative analysis of IBA1 fluorescence intensity(B), microglia density(C), CD68 intensity(D), microglia branch numbers(E) and branch lengths(F) in the RtTg of WT and shank3 knockout mice. Scale bars, 50 mm. Data are presented as means ± SEM. *P < 0.05; (WT, n = 8; KO, n = 9).

**Fig. 4:**
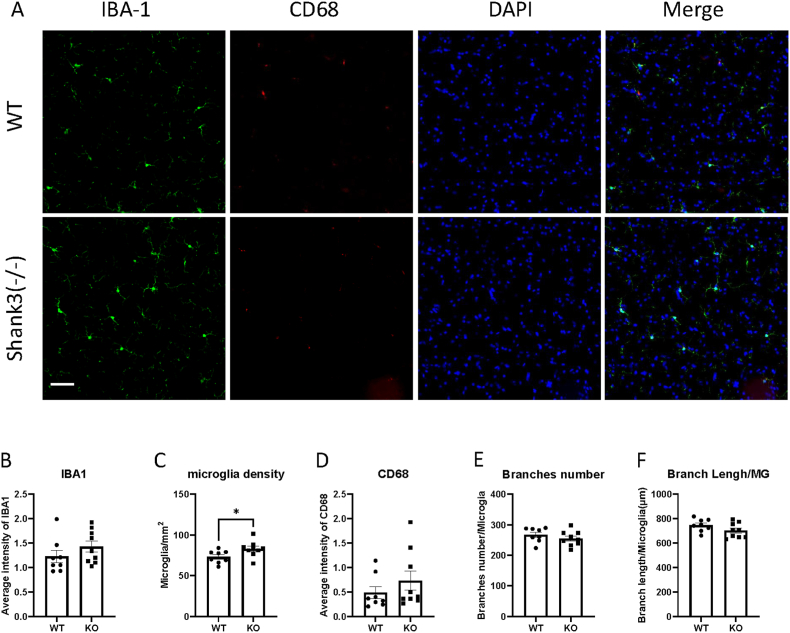
Shank3 knockout mice showed increased microglia density in PnC. (A) Representative images showing the IBA1 and CD68 expression of PnC in WT and shank3 knockout mice brain sections. (B to F) Quantitative analysis of IBA1 fluorescence intensity(B), microglia density(C), CD68 intensity(D), microglia branch numbers(E) and branch lengths(F) in the PnC of WT and shank3 knockout mice. Scale bars, 50 mm. Data are presented as means ± SEM. *P < 0.05; (WT, n = 8; KO, n = 9).

**Fig. 5:**
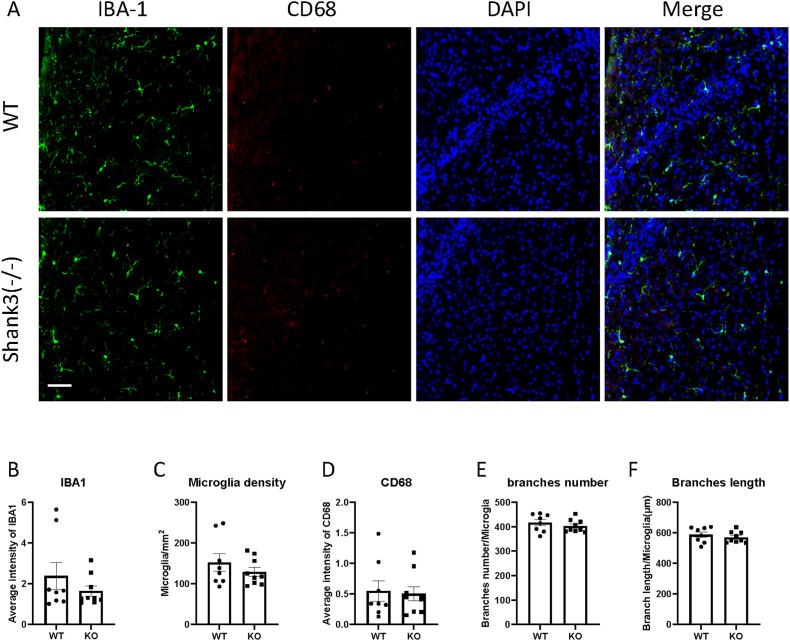
Shank3 knockout mice did not show microglia activation in CN. (A) Representative images showing the IBA1 and CD68 expression of CN in WT and shank3 knockout mice brain sections. (B to F) Quantitative analysis of IBA1 fluorescence intensity(B), microglia density(C), CD68 intensity(D), microglia branch numbers(E) and branch lengths(F) in the CN of WT and shank3 knockout mice. Scale bars, 50 mm. Data are presented as means ± SEM. *P < 0.05; (WT, n = 8; KO, n = 9).

**Fig. 6:**
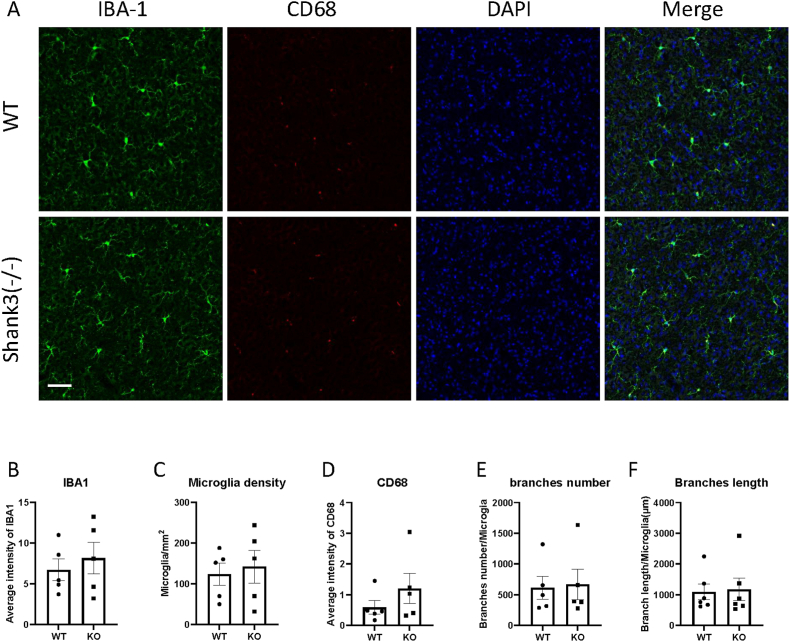
Shank3 knockout mice did not show microglia activation in IC. (A) Representative images showing the IBA1 and CD68 expression of IC in WT and shank3 knockout mice brain sections. (B to F) Quantitative analysis of IBA1 fluorescence intensity(B), microglia density(C), CD68 intensity(D), microglia branch numbers(E) and branch lengths(F) in the IC of WT and shank3 knockout mice. Scale bars, 50 mm. Data are presented as means ± SEM. *P < 0.05; (WT, n = 5; KO, n = 5).

To study the role of microglia in ASD, we measured microglial activation in Shank3 mice using immunofluorescence to assess the expression of activation-related markers such as IBA1. Fig. 1B illustrates the expression of IBA1 in CeA brain region. The results showed a significant increase in IBA1 expression in the CeA of Shank3 knockout mice compared to control mice (paired *t*-test, *t*(7) = 3.472, *p* = 0.0104, partial η^2^ = 0.63; mean difference = 1.314, 95% CI: 0.42–2.21), indicating that the CeA is more affected in Shank3 knockout mice. In contrast, no differences were observed in the somatosensory cortex (Fig. 2B), PnC (Fig. 3B), RtTg (Fig. 4B), CN (Fig. 5B), and IC (Fig. 6B) regions.

CD68 is another marker of microglial activation. Representative images showing CD68 and IBA1 immunofluorescent staining are presented in Fig. 1A–6A. We assessed CD68 expression by analyzing CD68 through immunofluorescent staining. Fig. 2D–6D shows the expression of CD68 in various brain regions. The fluorescence intensity of CD68 colocalized with IBA1 was measured. No significant differences in CD68 expression were observed between Shank3 and wild-type mice in any brain region, indicating no phagocytosis-related microglial activation in Shank3 mice.

An increased number of microglia is also indicative of microglial activation. To study microglial density, we counted the number of microglia after IBA1 staining. Fig. 4C illustrates the density of microglia in the PnC brain region. An increase in microglial density was observed in the PnC region of Shank3 mice compared to wild-type mice (paired *t*-test, *t*(7) = 2.919, *p* = 0.0224, partial η^2^ = 0.55). No differences were observed in other brain regions (Figs. 1C, 2C and 3C, 5C and 6C), suggesting increased microglial activation specifically in the PnC region of Shank3 knockout mice.

Microglia undergo morphological changes upon activation. To study the morphological properties of microglia in ASD, IBA1 was used to stain microglial morphology. We measured the total length and number of branches. Fig. 1E&F-6E&F illustrates the branch numbers and lengths of microglia in different brain regions. The results showed no significant differences in the morphological properties of microglia between wild-type and Shank3 mice, indicating no morphological activation of microglia in Shank3 mice.

## 4 Discussion

In summary, our results indicate that microglial activation in Shank3 knockout mice is region-specific. The CeA and PnC regions are notably affected in Shank3 knockout mice, while no significant changes in microglial activation were observed in the somatosensory cortex, RtTg, IC and CN regions. Mounting evidence indicates that activated microglia contribute to the pathogenesis of neurodegenerative diseases and neurodevelopmental disorders, such as Alzheimer's disease and ASD. Postmortem brain tissue from individuals with ASD has shown significant microglial activation [23]. Microglial activation is closely associated with neuronal function, influencing synaptic pruning and neuroinflammation [24]. Extensive studies have explored neural development and synapse formation in ASD, yet the direct link between microglial activation and neurodevelopmental alterations in ASD remains unclear. In this study, using the Shank3 ASD mouse model and fluorescence microscopy, we investigated microglial activation in brain regions related to auditory processing. Our findings demonstrate mild microglial activation in the CeA and PnC of Shank3 mice, with no significant activation observed in other brain regions. The CeA–PnC pathway plays a key role in modulating the acoustic startle reflex. The CeA sends projections to the PnC and can enhance startle responses under emotional or threat-related contexts. Therefore, the observed increase in IBA1 expression in the CeA and elevated microglial density in the PnC may reflect alterations in microglial regulation within this startle-modulating circuit in Shank3 mice. These results are important for understanding ASD pathogenesis and identifying potential therapeutic targets. This study offers valuable new insights into the role of microglia in ASD and highlights future research directions by coupling microglial alterations with brain regions responsible for auditory information processing.

The somatosensory cortex processes sensory inputs such as touch, temperature and pain. Changes in somatosensory pathways have been observed in children with ASD [25]. Recent studies support the role of the somatosensory cortex in gating auditory processing [26]. Our results show no changes in the expression of microglial activation markers IBA1 and CD68 in the somatosensory cortex of Shank3 knockout mice, nor were there significant morphological changes in microglia. These findings suggest that microglia in the somatosensory cortex do not exhibit noticeable activation in adult Shank3 ASD mice. The absence of microglial alterations in the somatosensory cortex suggests that microglial changes in Shank3 mice may be region-specific rather than reflecting global microglial activation across sensory cortices.

The acoustic startle response is commonly used to study auditory processing in mice. Several brain regions are involved in the acoustic startle response, starting from the cochlea. The cochlear nucleus, receiving auditory nerve input from the cochlea, is crucial for auditory processing, and dysregulated function in this region is linked to auditory processing disorders [27]. Our results indicate no changes in IBA1 and CD68 expression, nor in the number or morphology of microglia, in the cochlear nucleus of Shank3 knockout mice, suggesting no microglial activation in this region. The RtTg sends projections to the cochlear nucleus, and its neuronal activation is known to enhance the startle response [28]. Interestingly, no significant microglial alterations were detected in the cochlear nucleus, inferior colliculus (IC)or RtTg. This suggests that microglial changes in Shank3 mice may not occur uniformly across the auditory pathway but rather affect specific nodes involved in the modulation of the acoustic startle reflex. Our findings confirm the lack of microglial activation in the RtTg, as indicated by unchanged IBA1 and CD68 expression and no differences in microglial number or morphology in Shank3 knockout mice.

In the PnC region, we observed a significant increase in microglial cell density in Shank3 knockout mice compared to wild-type mice. This suggests potential microglial activation, yet no changes were detected in the morphology or expression of CD68 and IBA1. This indicates that some form of microglial activation in the PnC is independent of changes in CD68 and IBA1. The CeA in the amygdala sends direct projections to the PnC, regulating the startle response and modulating PPI levels [[29], [30], [31]]. Our results show increased IBA1 expression in the CeA, but no differences in CD68 expression, microglial number, or morphology between wild-type and Shank3 mice. These findings suggest that increased microglial activation in the CeA may contribute to the dysregulated auditory processing observed in ASD.

It remains unclear whether the microglial alterations observed in this study represent transient or persistent changes. Since the present experiments were performed in adult mice, future studies examining multiple developmental stages will be required to determine whether these changes arise during development or represent long-term alterations in microglial activity.

## 5 Conclusion

In summary, our data highlight region-specific microglial alterations in brain regions involved in the acoustic startle circuit, while no significant changes were observed in the somatosensory cortex used as a comparison region. We identified increased IBA1 expression in the CeA and a higher microglial density in the PnC of Shank3 knockout mice, suggesting disrupted CeA-PnC microglial activity in these mice. These results suggest a potential role of microglia in neural circuits underlying auditory startle responses in Shank3 mice.

## Funding

This work was supported by the China Scholarship Council (CSC).

## CRediT authorship contribution statement

**Xin Ren:** Conceptualization, Data curation, Formal analysis, Investigation, Methodology, Visualization, Writing – original draft. **Robbert Havekes:** Conceptualization, Supervision, Writing – review & editing. **Martien J.H. Kas:** Conceptualization, Project administration, Supervision, Writing – review & editing.

## Declaration of competing interest

The authors have no conflicts of interest.

## Data Availability

Data will be made available on request.

## References

[1] Persico A.M. (Aug 30 2021). The pediatric psychopharmacology of autism spectrum disorder: a systematic review - part I: the past and the present. Prog. Neuropsychopharmacol. Biol. Psychiatry.

[2] Han X. (Jan 2025). Deficiency of FABP7 triggers premature neural differentiation in idiopathic normocephalic autism organoids. Adv. Sci. (Weinh.).

[3] Hilal M.L., Rosina E., Pedini G., Restivo L., Bagni C. (May 2025). Dysregulation of the mTOR-FMRP pathway and synaptic plasticity in an environmental model of ASD. Mol. Psychiatr..

[4] Qing L. (Dec 2025). Maternal-infant probiotic transmission mitigates early-life stress-induced autism in mice. Gut Microbes.

[5] Trost B. (Nov 10 2022). Genomic architecture of autism from comprehensive whole-genome sequence annotation. Cell.

[6] Satterstrom F.K. (Feb 6 2020). Large-scale exome sequencing study implicates both developmental and functional changes in the neurobiology of autism. Cell.

[7] Durand C.M. (Jan 2007). Mutations in the gene encoding the synaptic scaffolding protein SHANK3 are associated with autism spectrum disorders. Nat. Genet..

[8] Lilja J. (Apr 2017). SHANK proteins limit integrin activation by directly interacting with Rap1 and R-Ras. Nat. Cell Biol..

[9] Duffney L.J. (Jun 9 2015). Autism-like deficits in Shank3-Deficient mice are rescued by targeting actin regulators. Cell Rep..

[10] Zhou Y. (Jan 6 2016). Mice with Shank3 mutations associated with ASD and schizophrenia display both shared and distinct defects. Neuron.

[11] Orefice L.L., Zimmerman A.L., Chirila A.M., Sleboda S.J., Head J.P., Ginty D.D. (Jul 14 2016). Peripheral mechanosensory neuron dysfunction underlies tactile and behavioral deficits in mouse models of ASDs. Cell.

[12] Zetler N.K., Cermak S.A., Engel-Yeger B., Baranek G., Gal E. (May 1 2022). Association between sensory features and high-order repetitive and restricted behaviors and interests among children with autism spectrum disorder. Am. J. Occup. Ther..

[13] Than A. (Jul 2024). Sensory over-responsivity and atypical neural responses to socially relevant stimuli in autism. Autism Res..

[14] Rendall A.R., Perrino P.A., Buscarello A.N., Fitch R.H. (Feb 2019). Shank3B mutant mice display pitch discrimination enhancements and learning deficits. Int. J. Dev. Neurosci..

[15] Goncalves A.M., Sousa N., Jacinto L., Monteiro P. (2023). The Shank3-InsG3680(+/+) mouse model of autism spectrum disorder displays auditory avoidance in a novel behavioral test. Front. Behav. Neurosci..

[16] Orefice L.L. (Aug 8 2019). Targeting peripheral somatosensory neurons to improve tactile-related phenotypes in ASD models. Cell.

[17] Huzard D. (2025/07/28 2025). Primary sensory neuron dysfunction underlying mechanical itch hypersensitivity in a Shank3 mouse model of autism. Transl. Psychiatry.

[18] Balasco L. (Jul 12 2022). Abnormal whisker-dependent behaviors and altered cortico-hippocampal connectivity in Shank3b-/- mice. Cerebr. Cortex.

[19] El-Cheikh Mohamad A., Mohrle D., Haddad F.L., Rose A., Allman B.L., Schmid S. (Oct 18 2023). Assessing the Cntnap2 knockout rat prepulse inhibition deficit through prepulse scaling of the baseline startle response curve. Transl. Psychiatry.

[20] Boyle C.A., Hu B., Quaintance K.L., Mastrud M.R., Lei S. (Oct 2022). Ionic signalling mechanisms involved in neurokinin-3 receptor-mediated augmentation of fear-potentiated startle response in the basolateral amygdala. J. Physiol..

[21] Albertini G. (Jun 2023). Serotonin sensing by microglia conditions the proper development of neuronal circuits and of social and adaptive skills. Mol. Psychiatr..

[22] Paxinos G., Franklin K.B.J. (2019).

[23] Lee A.S., Azmitia E.C., Whitaker-Azmitia P.M. (May 2017). Developmental microglial priming in postmortem autism spectrum disorder temporal cortex. Brain Behav. Immun..

[24] Wu X. (Oct 1 2023). Complement C1q drives microglia-dependent synaptic loss and cognitive impairments in a mouse model of lipopolysaccharide-induced neuroinflammation. Neuropharmacology.

[25] Failla M.D. (2017). Intrainsular connectivity and somatosensory responsiveness in young children with ASD. Mol. Autism.

[26] Godenzini L., Alwis D., Guzulaitis R., Honnuraiah S., Stuart G.J., Palmer L.M. (Jul 23 2021). Auditory input enhances somatosensory encoding and tactile goal-directed behavior. Nat. Commun..

[27] Balmer T.S., Trussell L.O. (Apr 20 2022). Descending axonal projections from the inferior colliculus target nearly all excitatory and inhibitory cell types of the dorsal cochlear nucleus. J. Neurosci..

[28] Guo W. (2021/11/04 2021). A brainstem reticulotegmental neural ensemble drives acoustic startle reflexes. Nat. Commun..

[29] Cano J.C., Huang W., Fénelon K. (Jun 3 2021). The amygdala modulates prepulse inhibition of the auditory startle reflex through excitatory inputs to the caudal pontine reticular nucleus. BMC Biol..

[30] Steinman M.Q. (Jul 2021). Importance of sex and trauma context on circulating cytokines and amygdalar GABAergic signaling in a comorbid model of posttraumatic stress and alcohol use disorders. Mol. Psychiatr..

[31] Boyle C.A., Lei S. (Jun 2023). Neuromedin B excites central lateral amygdala neurons and reduces cardiovascular output and fear-potentiated startle. J. Cell. Physiol..

